# Transcriptional Profiling of *C. albicans* in a Two Species Biofilm with *Rothia dentocariosa*

**DOI:** 10.3389/fcimb.2017.00311

**Published:** 2017-07-13

**Authors:** Priya Uppuluri, Henk J. Busscher, Jaideep Chakladar, Henny C. van der Mei, W. LaJean Chaffin

**Affiliations:** ^1^Division of Infectious Diseases, Los Angeles Biomedical Research Institute at Harbor-University of California Los Angeles Medical Center, Torrance Torrance, CA, United States; ^2^Department of Biomedical Engineering, University of Groningen and University Medical Center Groningen Groningen, Netherlands; ^3^Microbiology and Immunology, Texas Tech University Health Sciences Center Lubbock, TX, United States

**Keywords:** *Candida albicans*, *Rothia dentocariosa*, dual species, biofilm, voice prosthesis, laryngectomy

## Abstract

Biofilms on silicone rubber voice prostheses are the major cause for frequent failure and replacement of these devices. The presence of both bacterial and yeast strains has been suggested to be crucial for the development of voice prosthetic biofilms. Polymicrobial biofilms that include *Candida albicans* and *Rothia dentocariosa* are the leading cause of voice prosthesis failure. An *in vitro* biofilm comprising these two organisms was developed on silicone rubber, a material used for Groningen button voice prosthesis. We found that this biofilm environment was not conducive for *C. albicans* growth or differentiation. Global transcriptional analyses of *C. albicans* biofilm cells grown with *R. dentocariosa* revealed that genes with functions related to cell cycle progression and hyphal development were repressed >2-fold. The mixed species biofilms were more compact and less robust compared to *C. albicans* mono-species biofilms, even when developed under conditions of continuous nutrient flow. Under these conditions *R. dentocariosa* also significantly inhibited *C. albicans* biofilm dispersal. Preferential adherence of *R. dentocariosa* to candidal hyphae was mediated by the adhesin Als3.

## Introduction

Silicone rubber voice prostheses are used for rehabilitation of speech after total laryngectomy, and are inserted in a non-sterile habitat. As a consequence, these devices are found to be rapidly colonized by bacteria and yeasts, leading to deterioration of the voice prosthesis. While the functional lifetime of voice prostheses in patients, ranges from several days to years; this period is most often cut short due to rapid biofilm formation, with an average in situ lifetime of around 3 to 4 months (Van Den Hoogen et al., 1996; van Weissenbruch et al., 1997; Op de Coul et al., 2000; Buijssen et al., 2012). Biofilms formed on silicone rubber voice prosthesis, largely comprise of yeast and bacterial spp. and facilitate device deterioration, resulting in internal leakage, increased airflow resistance, and difficulties in respiration and swallowing (Sayed et al., 2012). Different clinical studies have identified the bacterial species *Rothia dentocariosa* and *Staphylococcus aureus* in biofilms on explanted prostheses of patients needing most frequent replacement, while *C. albicans* is one of the yeast generally held responsible for silicone rubber deterioration (Palmer et al., 1993; Elving et al., 2000). The *Rothia* spp. (*R. dentocariosa, R. aeria, R. nasimurium*, and *R. amarae*) are part of the normal flora of the human oropharynx and upper respiratory tract (Trivedi and Malhotra, 2015) and are commonly associated with dental caries and periodontal disease (Trivedi and Malhotra, 2015). Currently, this organism is considered as an emerging opportunistic pathogen and recent reports describe it as causing an array of life threatening diseases such as bacteremia (Ramanan et al., 2014; Abidi et al., 2016; Wang et al., 2016), endocarditis (Shands, 1988; Ruben, 1993), peritonitis (Morris et al., 2004; Keng et al., 2012), bone and joint infections (Trivedi and Malhotra, 2015), pneumonia (Wallet et al., 1997; Maraki and Papadakis, 2015), endophthalmitis (MacKinnon et al., 2001; Alvarez-Ramos et al., 2016), and prosthetic device infection (Elving et al., 2000, 2001, 2002, 2003; Millsap et al., 2001). Oropharyngeal infections due to adhering yeast and bacteria are responsible for a number of biomaterials-related infections, such as denture stomatitis (Radford et al., 1999) or malfunctioning of voice prosthesis in laryngectomized patients (Mahieu et al., 1986). In fact, *R. dentocariosa* and *C. albicans* are reported to be the predominant strains isolated from the mixed species biofilms responsible for replacement of voice prosthesis as early as 4 months of use (Elving et al., 2001, 2002). Besides *C. albicans, Candida tropicalis* has also been found to adhere in higher numbers to silicone rubber when adhering *R. dentocariosa, Lactobacillus* spp., or *S. aureus* is present (Millsap et al., 2001; van der Mei et al., 2014).

Processes involved in adhesion and biofilm formation by *C. albicans* have been investigated in considerable detail. Also, recent studies with biofilms containing *C. albicans* and bacterial species have suggested striking physiological interactions between the two adherent cell populations (Shirtliff et al., 2009). In several reports the bacterium associates with fungal hyphae. For example, within a biofilm, the opportunistic bacterial pathogen *Pseudomonas aeruginosa* is known to adhere, grow on, and kill only the *C. albicans* hyphae while surprisingly not able to attack the yeast form (Hogan and Kolter, 2002). *Streptococcus gordonii* adheres to hyphae which is mediated by the Candidal agglutinin like protein Als3 and bacterial SspB adhesins (Silverman et al., 2010) and biofilm development is influenced by bacterial signaling molecules (Bamford et al., 2009). Als3, which is the major adhesin of *C. albicans*, plays a key role in biofilm formation, host cell invasion and iron acquisition (Liu and Filler, 2011). This protein also mediates association of *S. aureus* with hyphae (Peters et al., 2012) which results in the alteration of protein profile of organisms in a dual species biofilm (Peters et al., 2010). Some studies on mixed species biofilm have focused on the consequence of this interaction on drug susceptibility patterns of both pathogens (Adam et al., 2002; Hogan and Kolter, 2002; Bamford et al., 2009; Diaz et al., 2012). For example, interactions with slime producing *S. epidermidis* enhances *C. albicans* resistance to fluconazole (Adam et al., 2002). We have initiated a study to investigate the interaction between two predominant pathogens responsible for silicone rubber voice prosthesis degradation, *C. albicans* and *R. dentocariosa*, in a biofilm setting. We have also extended the study to examine the impact of the presence of bacteria on *C. albicans* planktonic growth, biofilm dispersal and co-adhesion.

## Materials and methods

### Strains and media

The strains used in this study were *R. dentocariosa* GBJ 52/2B and *C. albicans* GBJ 13/4A, isolated from explanted silicone rubber voice prostheses, *C. albicans* SC5314 and *C. albicans als3/als3* (kindly provided by Dr. Stephen Saville) (Cleary et al., 2011). Stock cultures were stored in 15% glycerol in Yeast Peptone Dextrose (YPD) medium at −80°C. *R. dentocariosa* was routinely grown on Brain Heart Infusion agar (BD Biosciences, San Jose, CA), and incubated at 37°C. *C. albicans* were routinely grown on YPD agar plates and incubated at 30°C (0.5% yeast extract, 1% bacto peptone, 1% glucose).

### Mixed species static biofilm formation

Biofilms were developed on silicone rubber, the material used for Groningen button voice prostheses. *R. dentocariosa* GBJ 52/2B and *C. albicans* GBJ 13/4A were first grown overnight at 37 and 30°C respectively, on an agar plate from a frozen stock. One colony for the bacterial and yeast strains were used to inoculate a mixture of 30% brain heart infusion broth (OXOID, Basingstoke, Great Britain) and 70% defined yeast medium [per liter: 7.5 g glucose, 3.5 g (NH_4_)_2_SO_4_, 1.5 g L-asparagine, 10 mg L-histidine, 20 mg DL-methionine, 20 mg DL-tryptophane, 1 g KH_2_PO_4_, 500 mg MgSO_4_.7H_2_O, 500 mg NaCl, 500 mg CaCl_2_.2H_2_O, 100 mg yeast extract, 500 μg H_3_BO_3_, 400 μg ZnSO_4_.7H_2_O, 120 μg Fe(III)Cl_3_, 200 μg Na_2_MoO_4_.2H_2_O, 100 μg KI, 40 μg CuSO_4_.5H_2_O] and incubated at 37°C for 24 h. To develop a mixed species biofilm, a 1:2 ratio of *C. albicans* and *R. dentocariosa* pre-cultures were inoculated into fresh mixed medium, added to a glass container with a silicone rubber covered bottom and left for 5 h at 37°C. After 5 h, the silicone rubber was washed and fresh medium was added. The silicone rubber was incubated at 37°C for 24 h to promote mixed species biofilm development. A *C. albicans* mono-species biofilm was also cultured under similar conditions.

### Mixed species flow biofilm formation

Biofilms were developed under continuous media flow using the simple flow biofilm model, as described previously (Uppuluri and Lopez-Ribot, 2010). The medium used for the flow experiments was a 50:50 mixture of YPD (2% dextrose): BHI. This model involves a controlled flow (~1 ml/min, using a peristaltic pump) of fresh medium via Tygon tubing (Cole-Parmer, Vernon Hills, IL) into a 15 mL polypropylene conical tube (BD, Franklin, NJ) holding a 9 cm/1 cm silicone rubber (SR) strip. First, the autoclaved strips were pre-treated for 24 h with bovine serum. *C. albicans* and *R. dentocariosa* were grown overnight, washed, and incubated with the SR strips at a ratio of 1:10 (*C. albicans* 1 × 10^6^ cells/ml and *R. dentocariosa* 1 × 10^7^ cells/ml) for 5 h at 80 rpm agitation for the initial adhesion of cells. This 1:10 ratio of fungus:bacterium was chosen to achieve optimal adhesion of *R. dentocariosa* to fungal cells, prior to subjecting the cells to continuous flow of fresh medium that results in bacterial cells getting washed away over time. The flow process indirectly selects for the more robustly adhered bacterial populations. Next, the strip was inserted into the conical tube and the peristaltic pump was turned on. At various time points during biofilm development (2, 8, 12, and 24 h), cells spontaneously dispersed from the biofilm in the flow-through were collected from the bottom of the conical tube. We have previously reported (Uppuluri et al., 2010a) that *C. albicans* dispersed cells are predominantly yeast cells. The dispersed cells were quantified at each time point using a hemocytometer and compared (ANOVA *p* ≤ 0.05).

Biofilm biomass was quantified by measuring the dry weights of the biofilm. The total biomass of each biofilm was calculated by subtracting the weight of the silicone rubber prior to biofilm growth from the weight of the silicone rubber after biofilm growth, post dehydration of the biofilm containing SR strip for 16 h at 37°C. Statistically significant differences were analyzed using the *T*-test, with *p* < 0.05 considered statistically significant.

### *R. dentocariosa* and *C. albicans* planktonic growth conditions

*C. albicans* (1 × 10^5^ cells/ml) was co-inoculated with *R. dentocariosa* (1 × 10^5^ cells/ml and 1 × 10^6^ cells/ml) in YPD:BHI medium. Another flask of C. albicans cells were allowed to grow in the absence of *R. dentocariosa*, as control. The suspension was incubated overnight at 30°C, after which the cultures were diluted several fold and several aliquots were used to count yeast cells under a hemocytometer.

Next, *C. albicans-R. dentocariosa* co-cultures were monitored under hyphal permissive conditions. The fungus 1 × 10^6^ cells/ml (a cell number ideal for optimal hyphal induction) was mixed with the bacterium at a 1:10 and 1:100 ratio, in the 37°C pre-warmed liquid medium, buffered to pH 7. Cells were observed every hour for 7 h, under a bright field microscope and counted for proportion of yeast:germ tube/hyphal cells. *C. albicans* growth was also monitored in the presence or absence of *R. dentocariosa* conditioned medium. For conditioned medium preparation, *R. dentocariosa* 24 h cultures grown in YPD:BHI medium, were first centrifuged, and then media was filtered using filters with a 0.2 micron pore size. This cell-free medium was used for growing *C. albicans* yeast cells at three different starting concentrations 1 × 10^4^, 1 × 10^5^, and 1 × 10^6^ cells/ml. The conditioned medium was also supplemented with 2% glucose before *C. albicans* inoculation. For comparative controls, all three cell concentrations of *C. albicans* were grown in fresh media.

For some studies *C. albicans* 1 × 10^4^ cells/ml were grown in the cell-free medium diluted with fresh medium, to yield a final composition of 0, 25, 50, 75, and 100% conditioned medium.

### *R. dentocariosa* and *C. albicans* binding assay

*R. dentocariosa, C. albicans* SC5314, *C. albicans* GBJ 52/2B and a *C. albicans als3/als3* mutant were cultured for 24 h under their respective growth conditions. Bacteria were harvested by centrifugation (5,000 × g; 5 min), washed twice, and then suspended in several tubes of YPD:BHI medium at a concentration of 1 × 10^7^ cells/ml, and incubated at 37°C. After 1 h, respective *C. albicans* cells (1 × 10^6^) were added to individual tubes of this pre-warmed culture of *R. dentocariosa* and incubated further at 37°C for 6 h, to induce hyphal formation in the presence of the bacteria. This was done to serve two purposes, one, to study if presence of bacteria impaired *C. albicans* germ tube formation, and two, to find out the extent of bacterial binding to the fungus. Portions of the suspensions were then deposited onto microscope slides and visualized by light microscopy (40X magnification). At least 50 hyphal cells were counted for each co-binding pairings, from two independent experiments. The numbers of hyphae with clumps of bacterial binding vs. hyphae largely free of bacteria, were expressed as percentages of the total number of hyphae counted. The stability of adherence was followed by observing adherence after two gentle washes in PBS followed by centrifugation at 3,000 rpm for 5 min and re-suspension in sterile PBS.

### RNA extraction and cDNA synthesis

Cells from *C. albicans* mono species biofilm and mixed species biofilm were collected by scraping off the biofilm from the silicone rubber bottomed wells using a spatula. Total RNA was isolated using the standard hot acid phenol method, after grinding frozen cells in liquid nitrogen using a mortar and pestle (Uppuluri et al., 2007b). For control purposes, 24 h planktonic *R. dentocariosa* was also included for RNA extraction. The RNA preparation was DNAse-treated and the absence of *C. albicans* DNA contamination was confirmed with PCR amplification of the housekeeping gene *EFB1* (Maneu et al., 2000). RNA quantity was estimated spectrophotometrically at 260 nm and the RNA integrity verified by electrophoresis under non-denaturing condition in a 1.2% agarose gel, using Tris–acetate–EDTA buffer. The gel was stained with ethidium bromide (Sigma-Aldrich, St. Louis, MO) and observed under UV light. The amount of mRNA in the total RNA was quantified by using the Poly (A) mRNA detection system kit, (Promega, Madison, WI).

cDNA was synthesized using Oligo- 20 primer, 10 mM dNTP (includes AA-dUTP) mix and SuperScript III RT (Invitrogen, Carlsbad, CA) (Schmidt et al., 2002). The cDNA was labeled with Cy3 NHS ester (Amersham, Piscataway, NJ) and purified using the cDNA labeling and purification module (Invitrogen). Labelled cDNA was estimated spectrophotometrically at 550 and 650 nm.

### Transcriptional analyses

Corning UltraGAPS™II slides were printed with 70 mer oligonucleotides (QIAGEN, Valencia, CA) for approximately 6,000 *C. albicans* genes, by Oklahoma Medical Research Foundation Microarray Research Facility (Oklahoma City, OK). Labeled cDNA was hybridized on to the blocked microarray slides and washed at high stringency as previously described (Uppuluri et al., 2007a,b). Each condition was performed in quadruplicate. Slides were scanned with GenePix 4000B microarray scanner (Axon Instruments, Foster City, CA) and data was obtained using GenePix Pro 5.0 microarray image analysis software (Axon Instruments, East Lyme, CT). Analysis was performed with GeneSpring v7.2 software (Agilent Technologies, Santa Clara, CA). The expression level of some genes was very low and only genes with transcript levels at or above the 20th percentile were further analyzed. Cross-hybridization with *R. dentocariosa* was observed with 211 genes that were removed from further analysis (Supplementary Table S1). Differential gene expression of organisms recovered from the dual species biofilm compared to *C. albicans* biofilm alone was determined (*p* ≤ 0.05, *T*-Test with Benjamin-Hochberg multiple testing correction) and genes with at least a 2-fold change in expression were determined (*p* ≤ 0.05 *T*-test). The processes associated with differentially expressed genes were identified using Gene Ontology Slim Mapper (Inglis et al., 2012).

### Real time reverse transcriptase PCR (RTPCR)

RNA (1 μg) was treated with DNase I (Invitrogen, Carlsbad, CA) and followed by cDNA synthesis with the SuperScript® III RT enzyme (Thermoscientific, Grand island, NY) as per manufacturer's instructions. Expression levels of 23 genes were evaluated in this study. The genes were selected based on their expression changes in the mixed spp. environment vs. the single spp. growth. A total of 10 upregulated, 9 down regulated, and 4 unchanged genes were selected for validation of their expression patterns by this method. The primer sets were used in conjunction with SYBR Green PCR master mix and MicroAmp Fast Optical 96 well reaction plate (both ordered from Applied Biosystems), in an ABI PRISM 7,000 real-time PCR system (Applied Biosystems, Foster City, CA). Parameters for primer design were set according to the recommendations of Applied Biosystems, and as previously published by us (Uppuluri et al., 2010b). Briefly, the primer sizes were between 20 and 25 bases in length, and the *T*_*m*_ of each primer was 58°C. The amplicons were between 90 and 110 bp in size. Each reaction mixture was set up in triplicate in a 25 μl volume with 25 ng of cDNA for 40 cycles (thermal cycling conditions were initial steps of 50°C for 2 min and 95°C for 10 min, followed by 40 cycles of 95°C for 15 s and 60°C for 1 min). Relative gene expression was quantified using the threshold cycle (*C*_*T*_) method with the 7,300 System sequence detection software with the Relative Quantitation RQ study application from Applied Biosystems (Zhao et al., 2005). The target genes were normalized to the housekeeping gene *ACT1*. The variation in expression level was calculated for each biofilm by using the equation 2^−ΔΔ^*CT*, and results from the different replicates were averaged after the 2^−ΔΔ^*CT* calculations.

### Scanning electron microscopy

Biofilms of 24 h on silicone rubber were placed in fixative (4% formaldehyde v/v, 1% glutaraldehyde v/v in phosphate buffered saline) overnight. To preserve the integrity of biofilms and to minimize dehydration, the biofilms on silicone rubber were simply air dried in desiccators, coated with gold/palladium (40/60%) and observed in a scanning electron microscope (Leo 435 VP) in high vacuum mode at 15 kV. The images were processed for display using Photoshop software (Adobe, Mountain View, Calif.).

## Results

### Architecture of the *C. albicans*–*R. dentocariosa* mixed species biofilm

**Figure 1:**
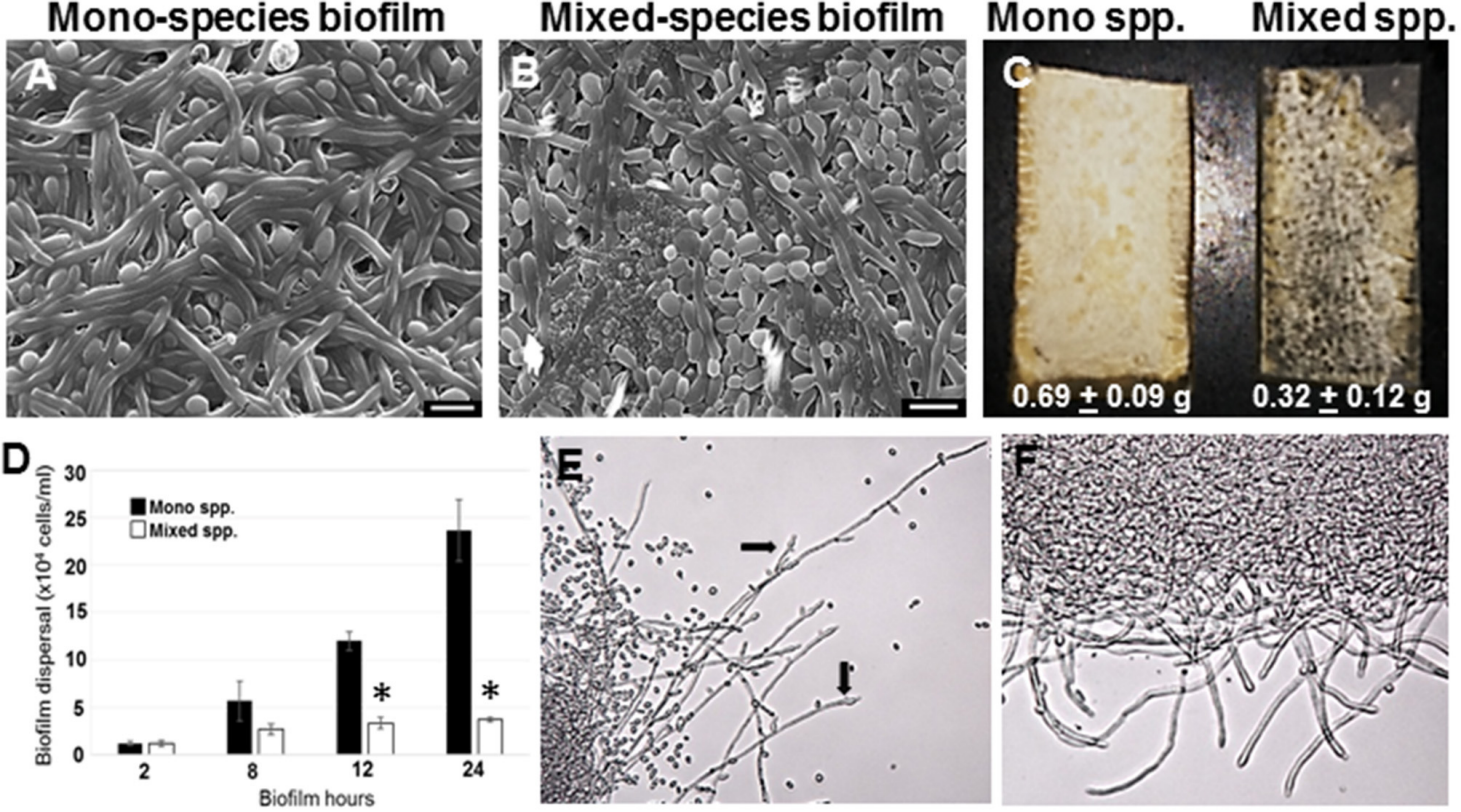
*C. albicans* biofilms were developed under static conditions on silicone rubber either in the absence **(A)** or presence of *R. dentocariosa* **(B)** and visualized by scanning electron microscopy (Mag: 1300X, scale bar: 10 μm). Biofilms were also developed in a flow system. *C. albicans* mono-spp. biofilms visually appeared denser than mixed spp. biofilms **(C)**, also indicated by the dry weights of the two biofilms under each biofilm. The frequency of *C. albicans* dispersal from both mono and mixed spp. biofilms were enumerated at different time points of biofilm formation. Asterisks identify a statistically significant *p*-value < 0.05, obtained by ANOVA test **(D)**. The top most hyphal layers of the biofilms grown under flow were examined for lateral yeast budding, under a light microscope (Mag: 20X)—Mono spp. flow biofilm **(E)** and Mixed spp. flow biofilm **(F)**.

*C. albicans* mono species as well as mixed species biofilms were grown on silicone rubber under static conditions for 24 h, and visualized using scanning electron microscopy (Figures 1A,B). While biofilms in the mono species biofilm presented an abundance of hyphae, a different picture emerged in the *C. albicans* biofilms containing *R. dentocariosa*. The mixed biofilms contained a higher number of yeast cells and pseudohyphae, compared to the *C. albicans* mono species biofilm. Visually, the mixed spp. biofilms appeared to be more compact and less robust compared to the *C. albicans* biofilm. The bacteria appeared to have occupied the empty channels created by hyphae in the biofilm. To estimate the impact of *R. dentocariosa* on *C. albicans* growth in the mixed biofilms, we enumerated the ratio of hyphal cells at the beginning (after 5 h of adhesion) and at the end of the 24 h biofilm, using a hemocytometer. While *C. albicans* mono species biofilm grew confluent hyphae after 24 h, we found no significant (*p* > 0.05) increase in the number of hyphal cells in the mixed species biofilm, indicating a stasis in *C. albicans* morphologic differentiation in the presence of *R. dentocariosa*. Additionally, we found that *C. albicans* cells in the mixed species biofilm were at least 11-fold less in number (1.3 × 10^7^ cells/ml) compared to the *C. albicans* mono-species biofilm (1.5 × 10^8^ cells/ml).

*R. dentocariosa* grows and multiplies more rapidly in media, compared to *C. albicans*. Thus, we questioned if nutrient starvation induced by *R. dentocariosa* or secondary metabolites secreted by this bacterium, could be the reason for reduced *C. albicans* growth and differentiation in the mixed biofilms. To test this hypothesis, we developed biofilms under conditions of continuous media flow, for 24 h. Both visual and dry weight measurements revealed at least a 2-fold reduction in the biomass of mixed biofilms compared to the *C. albicans* mono-species biofilms and this difference was found to be significant (*t*-test, *p* < 0.05) (Figure 1C). Interestingly, we found that despite the overall reduction in cell number under flow biofilm conditions, *C. albicans* hyphae in the mixed biofilm conditions under flow displayed elongated hyphae (Figure 1E).

### *C. albicans* dispersion from mixed species biofilms

Dispersion from biofilms is considered as the culprit causing biofilm mediated disseminated candidiasis. Since, *C. albicans* growing on voice prosthesis is often found in the presence of *R. dentocariosa*, we attempted to study the extent of dispersion of *C. albicans* cells from mixed biofilms vs. biofilm formed by the fungus alone. We collected cells released from biofilms at growth time-points ranging from 2 to 24 h. A statistically significant decrease (>4-fold, ANOVA *p* < 0.05) in *C. albicans* dispersal from the mixed spp. biofilms vs. mono-spp. biofilm was found only by 12 h, and this decrease in dispersal rose to 6-fold at 24 h (Figure 1D). Lateral yeast production by hyphae in *C. albicans* biofilms account for a majority of the dispersed cells. Microscopic imaging of the top-most layers of the biofilms indicated that *C. albicans* biofilms, as expected, demonstrated abundant lateral yeast production from the hyphal filaments (Figure 1E, arrows) and a drastic reduction in dispersal from mixed spp. biofilms (Figure 1F).

### Gene expression profiling of *C. albicans* recovered from a mixed species biofilm

**Figure 2:**
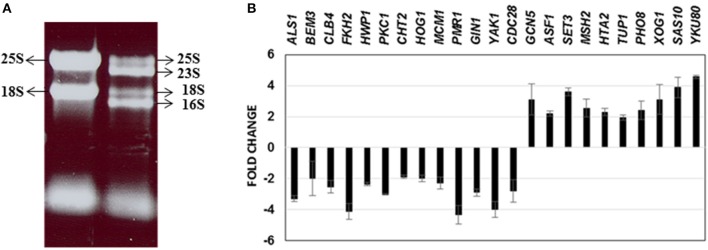
RNA obtained from *C. albicans* mono spp. as well as mixed spp. biofilms were separated on an agarose gel for visualization of RNA quality **(A)**. Real time PCR results of 23 *C. albicans* genes differentially regulated under mixed species biofilms (compared to mono spp. *C. albicans* biofilms) for validation of microarray gene expression patterns **(B)**.

Until date, little information is available on the genome-wide changes that occur in *C. albicans* because of its presence within a mixed species biofilm. We attempted to investigate the effect of mixed species association at a molecular level by analyzing the gene expression changes in *C. albicans* using microarray techniques. As a first step, RNA was extracted from the mono species as well as the mixed species biofilms as described earlier (Uppuluri et al., 2007b). RNA from mixed species biofilms resolved as four major bands indicating ribosomal sizes of 23S and 16S for bacterial rRNA and 25S and 18S for yeast rRNA (Figure 2A). As expected, the RNA from the yeast mono-species biofilms resolved as 2 major rRNA bands (Figure 2A). The total RNA extracted from both biofilm conditions was analyzed individually to determine the amount of messenger RNA (mRNA) present in each sample. This step ensured that regardless of the amount of the total RNA used as starting material for microarray hybridization, each sample contained equal amounts of mRNA. Inclusion of this step also rectified the possibility of contaminating bacterial RNA being estimated as a part of the total RNA, for subsequent experiments. Differential expression was observed for 1188 genes (*p* ≤ 0.05) A total of 633 *C. albicans* genes were found differentially regulated (317 up and 316 down) > 2-fold between mixed species and *C. albicans* only biofilm conditions (*p* ≤ 0.05) (Supplementary Table S2).

The data set of genes differentially regulated >2-fold were subjected to Gene Ontology analysis using the GO-Slim Mapper (Inglis et al., 2012), and clustered according to their biological processes. A brief summary of the differentially regulated processes along with their respective genes is presented in Table 1. The largest category of genes differentially regulated (37%) was of unknown functions. Genes involved in mitosis, maintenance of cell growth and proliferation (*BEM3, BNR1, AXL2, LAS1, CYB2, CLB4, CDC27, CDC28, HCM1, GIN1*) were down regulated >2-fold. Also downregulated were genes involved in cell wall biosynthesis and those encoding for GPI anchor proteins (*HOG1, ADA2, PKC1, HWP1, SSR1, TSC11, PMR1, GPI1, GPI17*). Several *C. albicans* genes playing a role in biofilm formation, e.g., *ALS1, CDR2, HWP1, MDR1, ZCF39*, were also downregulated in mixed biofilm conditions. A decrease in the transcript levels of cell wall biosynthesis genes and cell proliferation genes was in accordance with presented data that indicated reduced biomass, and a >11-fold decrease in cell number in the mixed species biofilm.

**Table 1:** List of *C. albicans* genes displaying up or down regulation in a mixed species biofilm with *R. dentocariosa*, after 24 h of incubation.

**Go term**	**Genes**
**DOWNREGULATED**	
Filamentous growth	*ADA2, ALS1, BEM3, CHT2, BNA4, CDS1, CHT2, CLB4, CTA4, ERG1, ERG13, FGR17, FGR24, FKH2, GIN1, HOG1, HWP1, MCM1, NBN1, PEP7, PMR1, RIM20, TEA1, TSC11, VID27, orf19.4459, orf19.5576*
Interspecies interaction	*ALS1, CTA4, CYB2, ENO1, FBA1, HSP70, RFX2, RIM20, orf19.7194*
Carbohydrate metabolism	*CDC43, CHS8, CHT2, DIE2, ENO1, FBA1, PMR1, PPG1, ROT2, orf19.3483, orf19.4611, orf19.7250, orf19.7426*
Transferase activity	*ARE2, CDC28, EPL1, GPI1, MCD4, MKK2, PKC1, PRI2, SAM2, SCT1, URK1, orf19.2281, orf19.3108, orf19.3466, orf19.4575, orf19.6581, orf19.6847, orf19.6980, orf19.7063, orf19.7140, orf19.7437*
GPI anchors	*CHT2, GA1, GPI1, GPI17, MCD4, PGA13, PGA27, PGA39, PGA48, SSR1, HWP1*
**UPREGULATED**	
RNA/DNA metabolism	*ALA1, ASF1, BRF1, CDC54, CAT8, CBF1, DCP2, DNA2, ESS1, GCN5, GSP1, HAS1, HBR1, HTA2, HTS1, IMP4, KRR1, LEA1, MDN1, MSH2, MSS116, NAN1, POL3, PRS, RAD10, RAT1, RNA1, RPB7, RRP15, SPT6, STI1, TEF2, TUP1, UTP18, UTP4, YKU80, ZCF1, orf19.1404, orf19.1491, orf19.2111, orf19.2262, orf19.2494, orf19.25, orf19.2795, orf19.2930, orf19.3161, orf19.3177, orf19.3220, orf19.3250, orf19.3303, orf19.4133, orf19.4634, orf19.4896, orf19.5297, orf19.5488, orf19.6259, orf19.6866, orf19.6923, orf19.7067, orf19.7097, orf19.7290, orf19.864, orf19.1730, orf19.211, orf19.2476, orf19.2579, orf19.4441, orf19.607, orf19.810*
Hydrolase activity	*BRO1, CNB1, DFG5, HAL22, INP51, KAR3, LKH1, MEF2, MLT1, NIT3, PHO8, PNC1, PRE9, PTC7, RPT5, SAP4, URA4, XOG1, YME1, YPT31, YPT7, org19.1887, orf19.1981, orf19.2026, orf19.2043, orf19.2249, org19.2285, org19.3437, orf19.354, orf19.522, orf19.607, orf19.6187, orf19.6742, orf19.7029, orf19.7216*
Vitamin metabolic process	*orf19.2590, orf19.3177, orf19.6057, orf19.6341*

Genes preferentially associated with hyphal development such as *HWP1, FKH2, MKK2, BEM3, YAK1* and *TUP1* were found differentially regulated >2-fold between the two biofilm conditions. Other major categories of hyphal genes such as those involved in the Ras-cAMP pathway (*EFG1, TPK1, TPK2*) or the MAP kinase pathway (*CEK1, CPH1, CST20*) were found to remain unchanged in the presence of *R. dentocariosa*. The absence of genes associated with morphogenesis may reflect that there are enough hyphae in the mixed species biofilm to not alter the global profile compared to a yeast cell only profile. Genes functioning in cell cycle regulation and cell wall biosynthesis contribute greatly toward morphogenetic functions and these were found downregulated in mixed spp. conditions.

**Figure 3:**
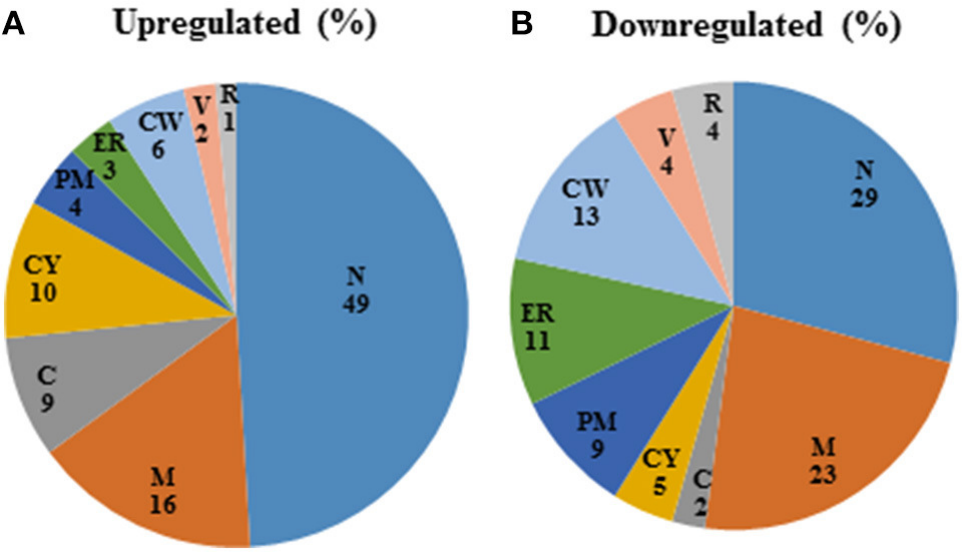
Pie-chart categorizing differentially expressed genes in the mixed spp. condition, into various cellular components. R, ribosome; V, vacuole; CW, cell wall; ER, endoplasmic reticulum; PM, plasma membrane; CY, cytoplasm; C, chromosome; M, mitochondria; N, nucleus.

Among those genes up-regulated in the mixed species biofilm, the majority had functions related to chromatin binding (*GCN5, MCM10*), chromatin silencing (*PNC1, SAS10*) and DNA damage repair (*YKU80, ASF1, MSH2, LCD1*). In fact >22% of the genes induced under mixed species biofilm condition were those involved in nucleic acid binding and metabolism (compared to only 11% in *C. albicans* mono species biofilm). This was also evident when the differentially regulated gene sets were categorized on basis of their cellular localization. Almost half of genes induced in the presence of *R. dentocariosa* localized to the nucleus (Figure 3A), with genes localizing to the chromosome, four times greater in number compared to the *C. albicans* biofilm alone. On the other hand, genes downregulated in the presence of the bacterium had a noteworthy presence (2–4 times more than the upregulated set) in the cell wall, the endoplasmic reticulum and the ribosome (Figure 3B).

We further corroborated our transcriptome results by quantitative real time PCR to analyze the expression of 23 *C. albicans* genes found either elevated or downregulated in the mixed spp. environment. Expression levels of all the 23 genes by PCR, mirrored the microarray data; amongst these were *ALS1, BEM3, CLB4, FKH2, CLB4, HWP1, PKC1, CHT2, HOG1, MCM1, PMR1, GIN1, YAK1*, and *CDC28—*genes involved in filamentation and biofilm growth, that were found downregulated. Results of the real time PCR study are summarized in Figure 2B.

### *C. albicans* planktonic growth and differentiation in the presence of *R. dentocariosa*

**Figure 4:**
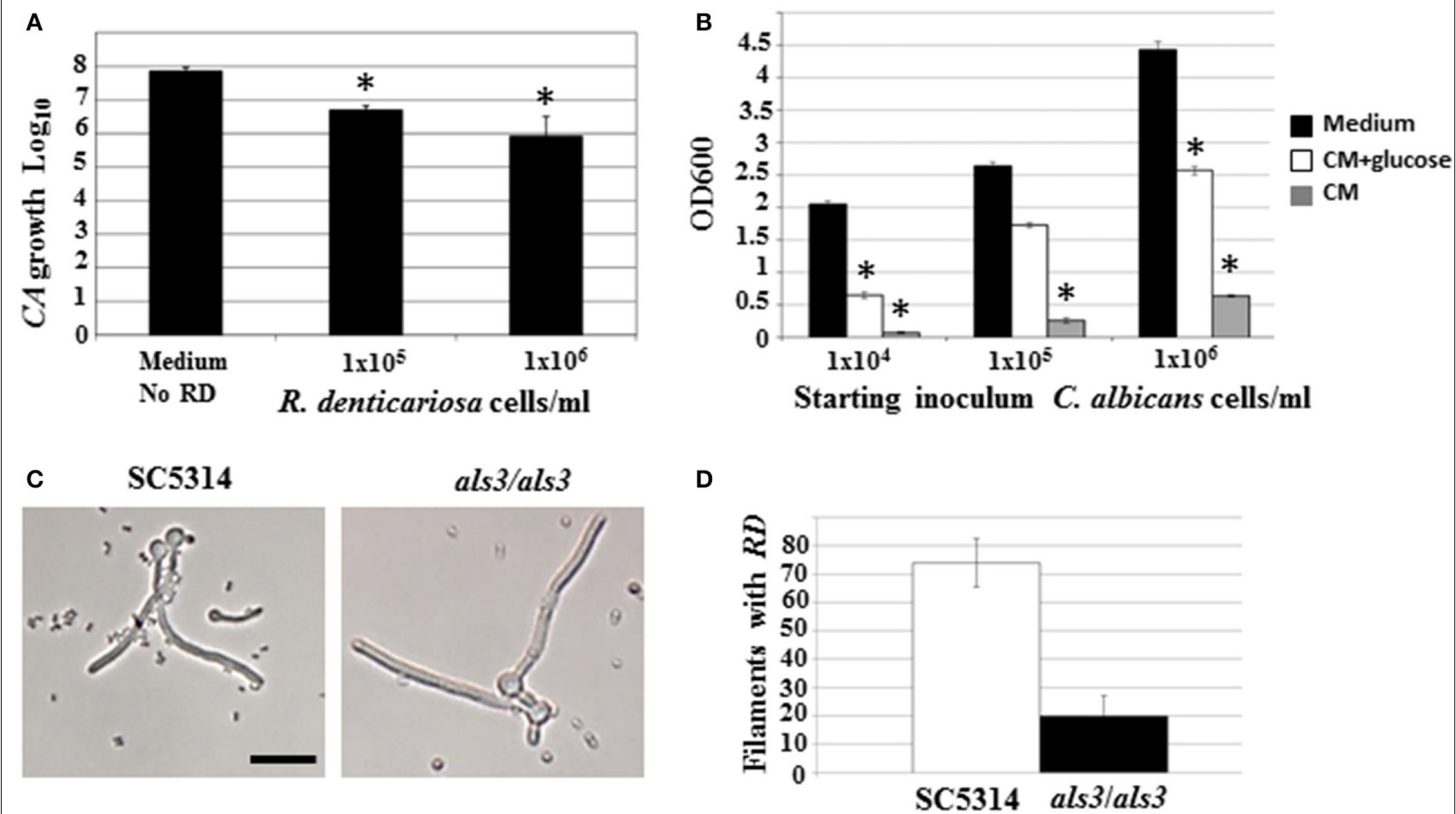
*C. albicans* yeast growth in the presence of different concentrations of *R. dentocariosa* was measured under planktonic conditions. Data was represented as Log_10_ of total cell numbers counted by a hemocytometer. The asterisks represent statistically significant reduction between all conditions (ANOVA, *p* < 0.05) **(A)**. Varying cell numbers of *C. albicans* were inoculated into control medium, and *R. dentocariosa* cell-free conditioned medium in the presence and absence of 2% glucose. Yeast growth after 24 h of incubation, was monitored by measuring OD600. The conditions that showed statistically significant reduction compared to the control (black bars) are denoted by an asterisk (ANOVA, *p* < 0.05) **(B)**. Interaction of *R. dentocariosa* with *C. albicans* SC5314 and *C. albicans* als3/als3 respectively, was visualized by light microscopy **(C)**, and the total number filaments of the two strains harboring *R. dentocariosa* were plotted **(D)**.

Both microarray as well as physiological studies demonstrated a reduction of *C. albicans* in the presence of *R. dentocariosa*, in a biofilm setting. We questioned to what extent such phenomenon was reflected in suspension culture. Hence, to investigate the dynamics of the mixed species interaction under planktonic conditions, *C. albicans* yeast cells and *R. dentocariosa* were inoculated at a ratio of 1:1, or 1:10 in suspension media and incubated at 30°C. After 24 h, a reduction between 10- and 50-fold in *C. albicans* cell number was observed which was directly proportional to the starting concentration of *R. dentocariosa* added to the mixture (Figure 4A).

We next studied the effect of *R. dentocariosa* on *C. albicans* differentiation. *C. albicans* at a concentration of 1 × 10^6^ cells/ml were added to a 10- to 100-fold concentrated *R. dentocariosa* culture suspension and incubated at 37°C. To rule out the effect of pH change due to microbial growth, the filamentation assay was done in media buffered to pH 7. Cells were observed every hour for 7 h, under a bright field microscope and counted for proportion of yeast:germ tube/hyphal cells. Surprisingly, even up to a cell number of 1 × 10^8^ cells/ml, *R. dentocariosa* did not have an inhibitory effect on *C. albicans* germ tube induction. However, >50% of the germ tubes were unable to undergo hyphal elongation after 3 h of incubation with *R. dentocariosa*. Interestingly, the bacterium appeared to adhere only to the hyphal filaments, and continued to do so even after a couple of gentle washes. Even after 10 h of interaction, the bacteria did not have an effect on the viability of the hyphal or yeast cells (data not shown).

### *C. albicans* planktonic growth and differentiation in the presence of *R. dentocariosa* conditioned medium

The mixed species biofilm environment yielded reduction in expression of genes involved in carbohydrate metabolic process, and induction of those enriched in the vitamin metabolic process. This indicated growth of the fungus in a milieu starved for nutrition. This was also noticed under planktonic growth conditions, where *C. albicans* exhibited growth retardation in the presence of *R. dentocariosa*. We postulated that this may be due to the depletion of nutrients by the rapidly growing bacterium, and the accumulation of secondary metabolites produced thereof. Hence, we investigated the influence of *R. dentocariosa* conditioned medium (cell-free growth medium) on *C. albicans* yeast growth and morphogenesis (Figure 4B). The growth medium alone (black bars) displayed maximum overnight proliferation of *C. albicans* yeast cells (range from 1 × 10^4^ to 1 × 10^6^ cells/ml starting concentration). Compared to that, *C. albicans* proliferation was drastically inhibited when grown in the bacterial conditioned medium (gray bars). In fact *C. albicans* at a starting concentration of 1 × 10^4^ cells/ml were inhibited >10-fold in the conditioned medium. To rule out the contribution of the lack of glucose in the conditioned medium toward this detrimental effect, we added glucose to the cell-free conditioned medium to a final concentration of 2%. Despite the presence of glucose (white bars), the conditioned medium inhibited planktonic yeast growth, ~2-fold compared to the control medium alone (Figure 4B). We additionally monitored *C. albicans* yeast growth in a mixture of fresh medium and varying concentrations of conditioned medium (0, 25, 50, 75, and 100%), with glucose levels always maintained at 2%. Conditioned medium at a concentration of 50% resulted in a 3-fold inhibition of planktonic yeast cells (1 × 10^4^ cells/ml), compared to growth in 0% conditioned medium.

### Role of a *C. albicans* adhesin als3 in *R. dentocariosa* binding

Our study revealed that *R. dentocariosa* adheres to *C. albicans* hyphal filaments. We questioned if *C. albicans* adhesins may contribute to this binding. We co-cultured *C. albicans* wild type strain (1 × 10^6^ cells/ml) along with *R. dentocariosa* (1 × 10^7^ cells/ml) under planktonic, hyphal permissive conditions. Another culture suspension under similar growth conditions was initiated with a mixture of *C. albicans als3/als3* mutant and *R. dentocariosa*. After 6 h of growth, we found that *R. dentocariosa* adhered to the wildtype *C. albicans* hyphae in large numbers, frequently growing as clusters on the filaments (Figure 4C). These clusters bound tightly and were resistant to gentle washes with PBS. On the other hand, the hyphal filaments of the *C. albicans als3/als3* mutants were largely bacteria-free (Figure 4C). Occasionally a few cells of *R. dentocariosa were* found attached to the hyphae, which easily detached after a couple of light PBS washes. Overall, at least 70% of the *C. albicans* wild-type hyphal filaments were found harboring *R. dentocariosa*, vs. only 20% of the *C. albicans als3*/*als3* hyphae that were bound by the bacterium (Figure 4D).

## Discussion

In the present study, we have investigated the interactions between *C. albicans* and *R. dentocariosa* in biofilms, as well as planktonic conditions, *in vitro*. Scanning electron microscopy of the biofilm and cell number enumeration experiments revealed that *C. albicans experienced* >10-fold growth retardation in the presence of *R. dentocariosa*, including a detrimental effect on hyphal proliferation as a result of the mixed species interaction (Figure 1). A recent report by van der Mei et al. (2014) on interactions between different *Candida* spp. with bacteria on silicone rubber revealed a similar loss in viability of *C. albicans* in the presence of *R. dentocariosa*. This report also observed a marginal induction in *C. albicans* hyphal formation in the presence of *R. dentocariosa*, a finding contrary to our static biofilm data that displayed an inertia in filamentous growth after 48 h. The report by Van der Mei developed biofilms under frequent changes in fresh media throughout the growth period. Our observations in the flow biofilm model resonated more with their findings, revealing that mixed spp. biofilms under flow conditions contained larger numbers of hyphal filaments (vs. in the static situation). However, in both flow and static biofilm conditions, the biomass of mixed biofilms was significantly smaller than the *C. albicans* mono spp. biofilms. Overall, this indicated that washing away of *R. dentocariosa* metabolites (in the flow system) perhaps had a positive impact on *C. albicans* differentiation. Nevertheless, sustained reduction in the biofilm biomass indicates a direct physical, detrimental influence of *R. dentocariosa* on *C. albicans* hyphal cells—a possibility confirmed under planktonic conditions. Keeping with the stasis in hyphal growth in the mixed spp. biofilm, we found a decrease in expression of some of the genes associated with filamentation and biofilm formation, such as *ALS1, YAK1, HWP1*, and *FKH2*.

Some interactions that cannot be easily identified under biofilm conditions, due to the close proximity of the two organisms with each other in high cell numbers and in a packed environment, can be clearly discovered under free-living growth conditions. For example, only under planktonic growth did we find that, *R. dentocariosa* appeared to adhere exclusively to *C. albicans* hyphal filaments and this adhesion was dependent on the *C. albicans* cell wall adhesin Als3 (Figures 4C,D). In fact, while >70% of wild type *C. albicans* hyphae had bound bacteria after 7 h of incubation, hyphae of an *als3*/*als3* mutant strain of *C. albicans* were virtually devoid of bacterial attachment. This pattern of preferential adherence has been illustrated earlier with *Pseudomonas aeruginosa* that not only adhered to, but also proved lethal to the *C. albicans* hyphae (Hogan et al., 2004; Morales et al., 2010). The contribution of adhesion molecules in yeast-bacterium attachment was first highlighted in a *S. gordonii* and *C. albicans* mixed biofilm model (Silverman et al., 2010). Several elegant assays pointed to the need for not only *Candida* adhesins (Als3) but also surface protein adhesins of the *Streptococcus* (SspA and SspB) for successful mutual adhesion.

In this study we found that *C. albicans* yeast growth under planktonic conditions was reduced between 10- and 50-fold on co-culture with *R. dentocariosa* (Figure 4A). We entertained the possibility of this occurrence due to early exhaustion of nutrients (especially glucose) from the growth media by the much rapidly growing *R. dentocariosa*. Growth in *R. dentocariosa* conditioned medium, supplemented with 2% glucose, and pH adjusted to 7.0, still had >2-fold deleterious effects on yeast growth, indicating factors other than nutrient limitation (perhaps quorum-sensing molecules secreted by *R. dentocariosa*) mediating the growth defect (Figure 4B).

This detrimental effect on *C. albicans* growth by *R. dentocariosa* was clearly manifested in the form of a much-reduced biofilm and reduced biofilm dispersal (Figure 1). Dispersal from biofilms is facilitated by the ability of hyphal cells to generate lateral yeast cells, and is prevented by depletion of the *SET3* histone deacetylase complex genes in the cells (Uppuluri et al., 2010a; Nobile et al., 2014). We found a complete disappearance of lateral yeast cells on the hyphae extending from the mixed spp. biofilms, while the *C. albicans*-only biofilms possessed abundant lateral yeast cells. Perhaps *R. dentocariosa* attachment to biofilm hyphae inhibited signaling pathways involved in lateral yeast production in *C. albicans*. Direct effects of bacterial signaling on yeast growth has been reported in the case of *S. gordonii*, which mediates regulation of several farnesol signaling genes in *C. albicans* such as Cek1p, Mkc1p, and Hog1p (Bamford et al., 2009); some of these were also downregulated under our mixed biofilm conditions. In fact, we also saw elevation in the *SET3* gene, which is contraindicative for biofilm dispersal (Nobile et al., 2014).

Finally, our study has endeavored to decipher the nature of interaction between the opportunistic human pathogenic fungus, *C. albicans* and an emerging opportunistic bacteria *R. dentocariosa*—an association that is the root cause for frequent failure of voice prosthesis in laryngectomized patients. Our results show a distinct dominance of the bacterium over the fungus, in most major aspects of its growth and differentiation, both under static or flow biofilm conditions. Our studies under planktonic conditions further suggest a role of both bacterial secondary metabolites as well as physical attachment, for this bacteria-favorable outcome. An integration of knowledge gained from our planktonic as well as biofilm studies, together with a better understanding of the molecular profiling within cross-kingdom interactions, will shed light on ways to manage biofilm infestation of medical devices.

## Author contributions

PU was involved in all stages of this study—experimental as well as manuscript writing; HB helped with development of mixed species biofilm formation and proof reading of the manuscript; JC performed the Real time RTPCR experiments; Hv helped with experimental design of the studies, resources and writing of the manuscript; WC helped with the experimental design of the studies, analysis of the microarray data, and writing the manuscript.

### Conflict of interest statement

The authors declare that the research was conducted in the absence of any commercial or financial relationships that could be construed as a potential conflict of interest.

## References

[B1] Abidi M. Z. Ledeboer N. Banerjee A. Hari P. (2016). Morbidity and mortality attributable to Rothia bacteremia in neutropenic and nonneutropenic patients. Diagn. Microbiol. Infect. Dis. 85, 116–120. 10.1016/j.diagmicrobio.2016.01.005 26906191

[B2] Adam B. Baillie G. S. Douglas L. J. (2002). Mixed species biofilms of *Candida albicans* and *Staphylococcus epidermidis*. J. Med. Microbiol. 51, 344–349. 10.1099/0022-1317-51-4-344 11926741

[B3] Alvarez-Ramos P. Del Moral-Ariza A. Alonso-Maroto J. M. Marín-Casanova P. Calandria-Amigueti J. M. Rodríguez-Iglesias M.. (2016). First report of acute postoperative endophthalmitis caused by Rothia Mucilaginosa after phacoemulsification. Infect. Dis. Rep. 8:6320. 10.4081/idr.2016.6320 27103973 PMC4815942

[B4] Bamford C. V. d'Mello A. Nobbs A. H. Dutton L. C. Vickerman M. M. Jenkinson H. F. (2009). *Streptococcus gordonii* modulates *Candida albicans* biofilm formation through intergeneric communication. Infect. Immun. 77, 3696–3704. 10.1128/IAI.00438-09 19528215 PMC2737996

[B5] Buijssen K. J. van der Laan B. F. van der Mei H. C. Atema-Smit J. van den Huijssen P. Busscher H. J.. (2012). Composition and architecture of biofilms on used voice prostheses. Head Neck 34, 863–871. 10.1002/hed.21833 21953690

[B6] Cleary I. A. Reinhard S. M. Miller C. L. Murdoch C. Thornhill M. H. Lazzell A. L.. (2011). *Candida albicans* adhesin Als3p is dispensable for virulence in the mouse model of disseminated candidiasis. Microbiology 157, 1806–1815. 10.1099/mic.0.046326-0 21436220 PMC3167918

[B7] Diaz P. I. Xie Z. Sobue T. Thompson A. Biyikoglu B. Ricke A.. (2012). Synergistic interaction between *Candida albicans* and commensal oral streptococci in a novel *in vitro* mucosal model. Infect. Immun. 80, 620–632. 10.1128/IAI.05896-11 22104105 PMC3264323

[B8] Elving G. J. van der Mei H. Busscher H. van Weissenbruch R. Albers F. (2003). Influence of different combinations of bacteria and yeasts in voice prosthesis biofilms on air flow resistance. Antonie Van Leeuwenhoek 83, 45–55. 10.1023/A:1022952712257 12755479

[B9] Elving G. J. van Der Mei H. C. Busscher H. J. van Weissenbruch R. Albers F. W. (2001). Air-flow resistances of silicone rubber voice prostheses after formation of bacterial and fungal biofilms. J. Biomed. Mater. Res. 58, 421–426. 10.1002/jbm.1037 11410901

[B10] Elving G. J. van der Mei H. C. Busscher H. J. van Nieuw Amerongen A. Veerman E. C. van Weissenbruch R.. (2000). Antimicrobial activity of synthetic salivary peptides against voice prosthetic microorganisms. Laryngoscope 110, 321–324. 10.1097/00005537-200002010-00027 10680938

[B11] Elving G. J. van der Mei H. C. Busscher H. J. van Weissenbruch R. Albers F. W. (2002). Comparison of the microbial composition of voice prosthesis biofilms from patients requiring frequent versus infrequent replacement. Ann. Otol. Rhinol. Laryngol. 111, 200–203. 10.1177/000348940211100302 11915880

[B12] Hogan D. A. Kolter R. (2002). Pseudomonas-Candida interactions: an ecological role for virulence factors. Science 296, 2229–2232. 10.1126/science.1070784 12077418

[B13] Hogan D. A. Vik A. Kolter R. (2004). A *Pseudomonas aeruginosa* quorum-sensing molecule influences *Candida albicans* morphology. Mol. Microbiol. 54, 1212–1223. 10.1111/j.1365-2958.2004.04349.x 15554963

[B14] Inglis D. O. Arnaud M. B. Binkley J. Shah P. Skrzypek M. S. Wymore F.. (2012). The Candida genome database incorporates multiple Candida species: multispecies search and analysis tools with curated gene and protein information for *Candida albicans* and *Candida glabrata*. Nucleic Acids Res. 40, D667–D674. 10.1093/nar/gkr945 22064862 PMC3245171

[B15] Keng T. C. Ng K. P. Tan L. P. Chong Y. B. Wong C. M. Lim S. K. (2012). *Rothia dentocariosa* repeat and relapsing peritoneal dialysis-related peritonitis: a case report and literature review. Ren. Fail. 34, 804–806. 10.3109/0886022X.2012.678208 22506572

[B16] Liu Y. Filler S. G. (2011). *Candida albicans* Als3, a multifunctional adhesin and invasin. Eukaryot. Cell 10, 168–173. 10.1128/EC.00279-10 21115738 PMC3067396

[B17] MacKinnon M. M. Amezaga M. R. MacKinnon J. R. (2001). A case of *Rothia dentocariosa* endophthalmitis. Eur. J. Clin. Microbiol. Infect. Dis. 20, 756–757. 10.1007/s100960100589 11757983

[B18] Mahieu H. F. van Saene H. K. Rosingh H. J. Schutte H. K. (1986). Candida vegetations on silicone voice prostheses. Arch. Otolaryngol. Head Neck Surg. 112, 321–325. 10.1001/archotol.1986.03780030085017 3942639

[B19] Maneu V. Martinez J. P. Gozalbo D. (2000). Identification of *Candida albicans* clinical isolates by PCR amplification of an EFB1 gene fragment containing an intron-interrupted open reading frame. Med. Mycol. 38, 123–126. 10.1080/mmy.38.2.123.126 10817228

[B20] Maraki S. Papadakis I. S. (2015). Rothia mucilaginosa pneumonia: a literature review. Infect. Dis. 47, 125–129. 10.3109/00365548.2014.980843 25664502

[B21] Millsap K. W. Bos R. van der Mei H. C. Busscher H. J. (2001). Adhesive interactions between voice prosthetic yeast and bacteria on silicone rubber in the absence and presence of saliva. Antonie Van Leeuwenhoek 79, 337–343. 10.1023/A:1012013101862 11816977

[B22] Morales D. K. Jacobs N. J. Rajamani S. Krishnamurthy M. Cubillos-Ruiz J. R. Hogan D. A. (2010). Antifungal mechanisms by which a novel *Pseudomonas aeruginosa* phenazine toxin kills *Candida albicans* in biofilms. Mol. Microbiol. 78, 1379–1392. 10.1111/j.1365-2958.2010.07414.x 21143312 PMC3828654

[B23] Morris S. K. Nag S. Suh K. N. Evans G. A. (2004). Recurrent chronic ambulatory peritoneal dialysis-associated infection due to *Rothia dentocariosa*. Can. J. Infect. Dis. Med. Microbiol. 15, 171–173. 10.1155/2004/823463 18159489 PMC2094970

[B24] Nobile C. J. Fox E. P. Hartooni N. Mitchell K. F. Hnisz D. Andes D. R.. (2014). A histone deacetylase complex mediates biofilm dispersal and drug resistance in *Candida albicans*. MBio 5, e01201–e01214. 10.1128/mbio.01201-14 24917598 PMC4056552

[B25] Op de Coul B. M. Hilgers F. J. Balm A. J. Tan I. B. van den Hoogen F. J. van Tinteren H. (2000). A decade of postlaryngectomy vocal rehabilitation in 318 patients: a single Institution's experience with consistent application of provox indwelling voice prostheses. Arch. Otolaryngol. Head Neck Surg. 126, 1320–1328. 10.1001/archotol.126.11.1320 11074828

[B26] Palmer M. D. Johnson A. P. Elliott T. S. (1993). Microbial colonization of Blom-Singer prostheses in postlaryngectomy patients. Laryngoscope 103, 910–914. 10.1288/00005537-199308000-00013 8361293

[B27] Peters B. M. Jabra-Rizk M. A. Scheper M. A. Leid J. G. Costerton J. W. Shirtliff M. E. (2010). Microbial interactions and differential protein expression in *Staphylococcus aureus*-*Candida albicans* dual-species biofilms. FEMS Immunol. Med. Microbiol. 59, 493–503. 10.1111/j.1574-695X.2010.00710.x 20608978 PMC2936118

[B28] Peters B. M. Ovchinnikova E. S. Krom B. P. Schlecht L. M. Zhou H. Hoyer L. L.. (2012). *Staphylococcus aureus* adherence to *Candida albicans* hyphae is mediated by the hyphal adhesin Als3p. Microbiology 158, 2975–2986. 10.1099/mic.0.062109-0 22918893 PMC4083660

[B29] Radford D. R. Challacombe S. J. Walter J. D. (1999). Denture plaque and adherence of *Candida albicans* to denture-base materials *in vivo* and *in vitro*. Crit. Rev. Oral Biol. Med. 10, 99–116. 10.1177/10454411990100010501 10759429

[B30] Ramanan P. Barreto J. N. Osmon D. R. Tosh P. K. (2014). Rothia bacteremia: a 10-year experience at Mayo Clinic, Rochester, Minnesota. J. Clin. Microbiol. 52, 3184–3189. 10.1128/JCM.01270-14 24951810 PMC4313135

[B31] Ruben S. J. (1993). *Rothia dentocariosa* endocarditis. West. J. Med. 159, 690–691. 8128689 PMC1022467

[B32] Sayed S. I. Datta S. Deore N. Kazi R. A. Jagade M. V. (2012). Prevention of voice prosthesis biofilms: current scenario and future trends in prolonging prosthesis lifetime. J. Indian Med. Assoc. 110, 175–178. 23029949

[B33] Schmidt H. Lissner R. Struff W. Thamm O. Karch H. (2002). Antibody reactivity of a standardized human serum protein solution against a spectrum of microbial pathogens and toxins: comparison with fresh frozen plasma. Ther. Apheresis 6, 145–153. 10.1046/j.1526-0968.2002.00347.x 11982956

[B34] Shands J. W. Jr. (1988). *Rothia dentocariosa* endocarditis. Am. J. Med. 85, 280–281. 3400712

[B35] Shirtliff M. E. Peters B. M. Jabra-Rizk M. A. (2009). Cross-kingdom interactions: *Candida albicans* and bacteria. FEMS Microbiol. Lett. 299, 1–8. 10.1111/j.1574-6968.2009.01668.x 19552706 PMC4406406

[B36] Silverman R. J. Nobbs A. H. Vickerman M. M. Barbour M. E. Jenkinson H. F. (2010). Interaction of *Candida albicans* cell wall Als3 protein with *Streptococcus gordonii* SspB adhesin promotes development of mixed-species communities. Infect. Immun. 78, 4644–4652. 10.1128/IAI.00685-10 20805332 PMC2976310

[B37] Trivedi M. N. Malhotra P. (2015). Rothia prosthetic knee joint infection. J. Microbiol. Immunol. Infect. 48, 453–455. 10.1016/j.jmii.2012.12.001 23357608

[B38] Uppuluri P. Chaturvedi A. K. Srinivasan A. Banerjee M. Ramasubramaniam A. K. Köhler J. R.. (2010a). Dispersion as an important step in the *Candida albicans* biofilm developmental cycle. PLoS Pathog. 6:e1000828. 10.1371/journal.ppat.1000828 20360962 PMC2847914

[B39] Uppuluri P. Lopez-Ribot J. L. (2010). An easy and economical *in vitro* method for the formation of *Candida albicans* biofilms under continuous conditions of flow. Virulence 1, 483–487. 10.4161/viru.1.6.13186 21178492 PMC3073357

[B40] Uppuluri P. Mekala S. Chaffin W. L. (2007a). Farnesol-mediated inhibition of *Candida albicans* yeast growth and rescue by a diacylglycerol analogue. Yeast 24, 681–693. 10.1002/yea.1501 17583896

[B41] Uppuluri P. Perumal P. Chaffin W. L. (2007b). Analysis of RNA species of various sizes from stationary-phase planktonic yeast cells of *Candida albicans*. FEMS Yeast Res. 7, 110–117. 10.1111/j.1567-1364.2006.00143.x 17311589

[B42] Uppuluri P. Pierce C. G. Thomas D. P. Bubeck S. S. Saville S. P. Lopez-Ribot J. L. (2010b). The transcriptional regulator Nrg1p controls *Candida albicans* biofilm formation and dispersion. Eukaryot. Cell 9, 1531–1537. 10.1128/EC.00111-10 20709787 PMC2950430

[B43] Van Den Hoogen F. J. Oudes M. J. Hombergen G. Nijdam H. F. Manni J. J. (1996). The Groningen, Nijdam and Provox voice prostheses: a prospective clinical comparison based on 845 replacements. Acta Otolaryngol. 116, 119–124. 10.3109/00016489609137724 8820362

[B44] van der Mei H. C. Buijssen K. J. van der Laan B. F. Ovchinnikova E. Geertsema-Doornbusch G. I. Atema-Smit J.. (2014). Voice prosthetic biofilm formation and Candida morphogenic conversions in absence and presence of different bacterial strains and species on silicone-rubber. PLoS ONE 9:e104508. 10.1371/journal.pone.0104508 25111806 PMC4128802

[B45] van Weissenbruch R. Bouckaert S. Remon J. P. Nelis H. J. Aerts R. Albers F. W. (1997). Chemoprophylaxis of fungal deterioration of the Provox silicone tracheoesophageal prosthesis in postlaryngectomy patients. Ann. Otol. Rhinol. Laryngol. 106, 329–337. 10.1177/000348949710600413 9109726

[B46] Wallet F. Perez T. Roussel-Delvallez M. Wallaert B. Courcol R. (1997). *Rothia dentocariosa*: two new cases of pneumonia revealing lung cancer. Scand. J. Infect. Dis. 29, 419–420. 10.3109/00365549709011841 9360260

[B47] Wang J. Y. Brossard J. Cellot S. Dix D. Feusner J. Johnston D. L.. (2016). Invasive Rothia infections in children with acute myeloid leukemia: a report from the Canadian infections in AML research group. Pediatr. Hematol. Oncol. 33, 277–281. 10.1080/08880018.2016.1187231 27315594

[B48] Zhao R. Daniels K. J. Lockhart S. R. Yeater K. M. Hoyer L. L. Soll D. R. (2005). Unique aspects of gene expression during *Candida albicans* mating and possible G(1) dependency. Eukaryot. Cell 4, 1175–1190. 10.1128/EC.4.7.1175-1190.2005 16002644 PMC1168966

